# Supplementary material for: Integration of molecular coarse-grained model into geometric representation learning framework for protein-protein complex property prediction
Source: Nat Commun. 2024 Nov 7;15:9629. doi: 10.1038/s41467-024-53583-w (PMC11544137; doi:10.1038/s41467-024-53583-w)
Supplement: Supplementary file 1 — Supplementary Information [file 41467_2024_53583_MOESM1_ESM.pdf]

Supplementary Information (SI)

Integration of molecular coarse-grained model into geometric representation learning framework for protein-protein complex property prediction

Yang Yue[1], Shu Li[2], Yihua Cheng[1], Lie Wang[3], Tingjun Hou[4], Zexuan Zhu[5*], Shan He[1,2*]

[1]School of Computer Science, The University of Birmingham, Edgbaston, Birmingham, B15 2TT, UK. [2]Centre of Artificial Intelligence driven Drug Discovery, Faculty of Applied Science, Macao Polytechnic University, Macao SAR 999078, China. [3]Bone Marrow Transplantation Center of the First Affiliated Hospital, and Institute of Immunology, Zhejiang University School of Medicine, Hangzhou, 310058, China. [4]College of Pharmaceutical Sciences, Zhejiang University, Hangzhou, 310058, China. [5]National Engineering Laboratory for Big Data System Computing Technology, Shenzhen University, Shenzhen, 518060, China. Yang Yue and Shu Li contributed equally to this work. Correspondence and requests for the material should be addressed to Z.Z. and S.H. (email: zhuzx@szu.edu.cn and s.he@cs.bham.ac.uk)

The statistics of graph nodes and edges of the MANY/DC dataset

Based on the standard dimer dataset MANY/DC[1,2] and conventional dataset splitting settings[3,4] as an example, we give the statistics information of nodes and edges of the protein complex graphs at different scales, in which the atom- and residue-scale complex graphs are constructed based on the default settings of atom- and residue-scale GearNet-Edge (i.e., the counterparts of MCGLPPI), respectively[5,6]. Additionally, to differentiate the results of MCGLPPI supported by MARTINI22 and MARTINI3, we denote their corresponding scale names as "CG-M2" and "CG-M3", respectively (Supplementary Table 1).

Supplementary Table 1: the statistics of nodes and edges in each set of the MANY/DC dataset

**Scale**	**Sub-set**	**Graph node number**	**Graph edge number**	**Average Node degree**
CG-M2	Training	321.780	2373.738	7.377
CG-M2	Validation	321.315	2371.637	7.381
CG-M2	Test	392.377	2900.821	7.393
**Scale**	**Sub-set**	**Graph node number**	**Graph edge number**	**Average Node degree**
CG-M3	Training	341.764	2697.742	7.894
CG-M3	Validation	338.694	2669.420	7.882
CG-M3	Test	416.216	3298.975	7.926
**Scale**	**Sub-set**	**Graph node number**	**Graph edge number**	**Average Node degree**
Atom	Training	1226.182	18,897.831	15.412
Atom	Validation	1221.720	18,807.455	15.394
Atom	Test	1504.132	23,402.179	15.559
**Scale**	**Sub-set**	**Graph node number**	**Graph edge number**	**Average Node degree**
Residue	Training	156.996	3023.984	19.261
Residue	Validation	156.553	3016.193	19.266
Residue	Test	192.682	3772.550	19.579

We can first observe that, compared with the atom-scale counterpart based on the full-atom characterizations, the CG-scale protein complex graph produced by MCGLPPI has overall smaller graph size: the number of nodes and edges of the atom-scale graph, are approximately three times and seven times greater, respectively, than those at the CG-scale graph.

Meanwhile, for the residue-based counterpart, its modern design of graphs for protein

property predictions like residue-scale GearNet-Edge is more relying on the construction of edges, which are usually fully built based on multiple pre-defined geometric distance and sequential thresholds, aiming to capture more comprehensive spatial relationships between residue nodes[5-7]. While the number of edges will significantly influence the neighboring message aggregation[8] speed of corresponding graph neural networks. In contrast, our CG graph adopts more chemical-plausible edges according to the MARTINI force field. For these MARTINI-based edges, they are constructed based on the specific interaction definitions between designated bead node pairs, thereby reducing the reliance on indiscriminately wiring every node pair within multiple pre-defined thresholds. Under current settings, although the average graph node number in CG-scale is relatively higher, the average graph edge number and node degree are both observed a significant decrease, which is ultimately beneficial to overall more sparse and accurate edge distribution and thus contributes to effectively decreasing the processing speeds.

Besides, due to the moderate extension of bead types and numbers of MARTINI3, its corresponding graph size is slightly larger than that from MARTINI22.

The other experimental results for the evaluation of the DDI-based pre-training technique

Following the same experimental settings described in **The investigation of CG-scale pre-training techniques on different tasks** section of the manuscript, we here provide the predictive performance of the pre-trained MCGLPPI-M2, MCGLPPI-M3, and its atom- and residue-scale counterparts, which are evaluated based on the other

metrics (except for R_P and AUPR) on the downstream datasets.

From the experimental results (Supplementary Table 2), we find that under the current pre-training settings and evaluation metrics, the MCGLPPI-M2 and MCGLPPI-M3 still outperform their atom- and residue-scale counterparts, which is in line with the results shown in Fig. 3a of the manuscript.

In addition, under the full set of 3DID pre-training set, for MCGLPPI(-M2), the R_P on the PDBbind dataset, R_P on the ATLAS dataset, and AUPR on the MANY/DC dataset are 0.610, 0.836, and 0.865, respectively.

Supplementary Table 2: the other experimental results of the pre-training at different scales

**PDBbind**				
**Pre-training set**	**Model name**	**Scale**	**RMSE**	**MAE**
Complete 3did	MCGLPPI-M2	CG	**2.037**	1.572
The 33144-set	MCGLPPI-M2	CG	2.053	1.588
The 33144-set	MCGLPPI-M3	CG	2.048	**1.569**
The 33144-set	GearNet-Edge	Atom	2.163	1.646
The 33144-set	GearNet-Edge	Residue	2.165	1.632
**ATLAS**				
**Pre-training set**	**Model name**	**Scale**	**RMSE**	**MAE**
Complete 3did	MCGLPPI-M2	CG	**0.998**	0.765
The 33144-set	MCGLPPI-M2	CG	1.002	**0.745**
The 33144-set	MCGLPPI-M3	CG	1.024	0.778
The 33144-set	GearNet-Edge	Atom	1.052	0.771
The 33144-set	GearNet-Edge	Residue	1.056	0.808
**MANY/DC**				
**Pre-training set**	**Model name**	**Scale**	**AUROC**	
Complete 3did	MCGLPPI-M2	CG	0.874	
The 33144-set	MCGLPPI-M2	CG	**0.877**	
The 33144-set	MCGLPPI-M3	CG	0.860	
The 33144-set	GearNet-Edge	Atom	0.838	
The 33144-set	GearNet-Edge	Residue	0.855	

The bold data indicates the best experimental result under current dataset and evaluation metric.

The experimental results on more challenging experimental settings

Based on the PDBbind dataset, we conduct an additional more challenging experiment based on further structural homology reduction. Specifically, for the PDBbind 915-subset for which all involved methods can completely identified, we perform the pairwise TM-align structural alignments[9] against all samples in this 915-

subset, resulting in a 915×915 TM-score matrix with each element representing the structural similarity between the corresponding samples. Furthermore, for each sample, we choose the maximum TM-score in the corresponding row of the matrix, i.e., its maximum structural similarity against all other samples in the dataset. On top of this, we extract samples with maximum TM-score lower than 0.45 as the test set, representing the samples with the lowest homology structure similarities (with other all samples) in the dataset (124 samples in total). The samples with the TM-score ranging from 0.45-0.55 are used as the validation set (85 samples in total), and the rest of samples are treated as the training set (706 samples).

Supplementary Table 3: the experimental results based on structural homology reduction settings

**W/ pre-training**	**Scale**	$\boldsymbol{R}_{\mathbf{P}}$	**RMSE**	**MAE**
MCGLPPI-M2	CG	**0.449**	**2.084**	**1.666**
MCGLPPI-M3	CG	0.442	2.503	1.966
GearNet-Atom[6]	Atom	0.384	2.287	1.817
GearNet-Res[5]	Residue	0.369	2.370	1.857
**W/O pre-training**	**Scale**	$\boldsymbol{R}_{\mathbf{P}}$	**RMSE**	**MAE**
MCGLPPI-M2	CG	0.406	**2.111**	**1.723**
MCGLPPI-M3	CG	**0.426**	2.237	1.745
GearNet-Atom[6]	Atom	0.346	2.276	1.771
GearNet-Res[5]	Residue	0.365	2.541	2.037
GVP-GNN[10]	Atom	0.221	3.835	3.231

The bold data signifies the best experimental result under the current comparison group.

Based on the aforementioned experimental settings, we select the best basic model settings under the corresponding tenfold cross-validation (CV) settings for each involved method (including those with and without pre-training), to conduct the comparison (Supplementary Table 3). The experimental results indicate that, MCGLPPI-M2 and MARTINI-M3 brings significant performance improvements on Pearson's correlation coefficient (R_{P}) under this challenging test scenario, further

demonstrating the generalization ability of the proposed framework.

Model stability test using more accessible AlphaFold-generated structures

To examine the binding affinity prediction stability of models in the scenario in which the expensive real structures are not available, based on the PDBbind dataset, we conduct an extra experiment as follows.

We first generate the protein complex structures for samples in the PDBbind-strict-dimer dataset using the publicly available ColabFold[11], a simplified version based on AlphaFold[12] and AlphaFold-multimer[13]. The FASTA sequence file containing the complete sequences of both proteins in each dimer is used as input for the ColabFold. Following the ColabFold pipeline, we perform a MSA search with MMseqs2 on the UniRef30 database and generate five models for each sample. These models are subsequently relaxed with the Amber program and ranked. We ultimately select the top-ranked structure as the AlphaFold generated structure for each sample in the PDBbind-strict-dimer dataset. Then, the generated complex structures are transformed into the CG-graphs, and their atom-scale and residue-scale counterparts.

After that, 911 of 1270 samples are successfully identified by the atom- and residue-scale GearNet-Edge (which are also covered by the PDBbind 915-subset). Based on this 911-subset, we perform the comparison based on two different sample splitting settings, i.e., the standard tenfold CV and the TM-score-based splitting (702-85-124 samples for training, validation, test sets, detailed description is provided in the last section), and record the experimental results based on the best model configurations

under the real structure testing (Supplementary Table 4).

Supplementary Table 4: the experimental results based on AlphaFold-generated structures

**10-fold CV**	**Scale**	$\boldsymbol{R_P}$	**RMSE**	**MAE**
MCGLPPI-M2	CG	0.589	2.088	1.572
MCGLPPI-M3	CG	**0.599**	**2.084**	**1.566**
GearNet-Atom[6]	Atom	0.526	2.331	1.731
GearNet-Res[5]	Residue	0.567	2.156	1.623
**TM-score splitting**	**Scale**	$\boldsymbol{R_P}$	**RMSE**	**MAE**
MCGLPPI-M2	CG	0.404	**2.130**	**1.736**
MCGLPPI-M3	CG	**0.409**	2.970	2.378
GearNet-Atom[6]	Atom	0.292	2.249	1.803
GearNet-Res[5]	Residue	0.363	2.261	1.760

The bold data signifies the best experimental result under the current comparison group.

We observe that, under current experimental settings, the models trained with more accessible AlphaFold-generated structures achieve close predictive accuracy compared with those derived from real structures, indicating that when feeding the AlphaFold-generated structures, the models are also capable of adapting to their structural patterns to give reasonable predictions, which is valuable when the expensive real structures are difficult to acquire.

Further extension to protein complex ΔΔG predictions based on simple modifications

we demonstrate the potential of our method MCGLPPI on the extension into directly predicting ΔΔG with the simple modifications. Specifically, we use a multiple-point mutation dataset AB-bind[14], which contains 1101 sample points related to the binding affinity change (i.e., ΔΔG) caused by multiple-point AA mutations on the complex formed from antibody or antibody-like binding. Following the basic experimental settings of the existing pre-trained GNN-based approach MpbPPI[15], which has already tested on this dataset, FoldX[16] is chosen to complete all side chains and generate the

mutation complex structure based on the corresponding raw PDB file of wild-type (WT) structure and mutational site information (and then all structures will be transformed into CG-scale protein graphs based on MARTINI). Based on this, two groups of different validation experiments are conducted as follows.

For the first group of experiments, we first consider a WT protein-protein complex type-based fivefold cross-validation (CV) evaluation setting. This setting reduces the sample similarity between training and test sets by ensuring no intersection of original WT protein-protein complex types (for corresponding WT-mutant (MT) pairs) between any of the two folds and striving to make the both sample total number and WT protein-protein complex type number assigned for each fold to be as close as possible (detailed in original MpbPPI paper[15]). For the related model modification details, ΔΔG represents the binding affinity (i.e., binding free energy, ΔG) change from WT to MT status (i.e., $\Delta G^{WT} - \Delta G^{MT}$). Inspired by this[15,17], keeping other model configurations unchanged, the ΔΔG calculation is training optimized and then predicted based on a three-layer multi-layer perception (MLP) which receives the difference between graph embeddings of the CG complex graph before mutation and after mutation (i.e., $\mathbf{h}^{WT} - \mathbf{h}^{MT}$) provided by the protein encoder initiailized from the pre-trained model parameter checkpoint.

We evaluate our method (MCGLPPI-M2), and record the prediction results based on the R_P, RMSE, and MAE. We also report the corresponding results of MpbPPI and two representative energy-based specialized methods FoldX and Rosetta macromolecular modeling suite (Flex ddG)[18] from ref[15] (Supplementary Table 5).

From these experimental results, we find that under current settings, our method achieves competitive performance compared with the advanced ΔΔG prediction methods. It is worth noting that our method only adopts a straightforward adaptation without any specifically designed modules, while the compared methods are carefully proposed for the ΔΔG predictions.

Supplementary Table 5: the ΔΔG prediction results based on the AB-bind dataset

**5-fold WT type-based CV**	$\boldsymbol{R_P}$	**RMSE**	**MAE**
MCGLPPI	**0.451**	**1.880**	1.363
MpbPPI[15]	0.442	1.899	**1.357**
FoldX[16]	0.273	3.429	2.278
Flex ddG[18]	0.059	4.494	2.875
**TM-score splitting**	$\boldsymbol{R_P}$	**RMSE**	**MAE**
MCGLPPI	0.415	1.327	1.081
**AB-bind 962-subset inference**	$\boldsymbol{R_P}$	**RMSE**	**MAE**
MCGLPPI	**0.280**	**2.420**	**1.563**
GearNet-Atom[6]	0.150	2.464	1.606
GearNet-Res[5]	0.074	2.476	1.609
**AB-bind complete inference**	$\boldsymbol{R_P}$	**RMSE**	**MAE**
MCGLPPI	0.264	2.386	1.511

The bold data signifies the best experimental result under the current comparison group.

At the same time, based on the same model modifications, we further test the stability of our method through a data split with the explicit TM-score cutoff. Specifically, we utilize the TM-align alignment tool to calculate a similarity matrix based on the structural difference between pairwise PDB samples within AB-bind, and extract sample points whose TM-score is lower than 0.5 with other PDB samples as the test set (183 sample points are found in total), and the remaining ones are treated as the training set. In this setting, MCGLPPI still can give a test R_P over 0.4 (Supplementary Table 5).

On top of this, another group of experiments are conducted about investigating the

feasibility of directly using the trained ΔG models for ΔΔG predictions according to the existing data. Specifically, we train MCGLPPI-M2, GearNet-Atom, and GearNet-Res based on the 915-sample-subset of the independent PDBbind-strict-dimer ΔG dataset (see **The binding affinity prediction of formation of strict dimers** section of the manuscript), and predict ΔΔG for all samples in the 1101-sample AB-bind dataset based on the prediction of ΔG^{WT} minus the prediction of ΔG^{MT} without extra training (Supplementary Table 5, please note that for GearNet-Atom and GearNet-Res, 962 out of all test samples can be identified and inferred). We observe that, although our model is trained with an independent ΔG dataset that does not include specialized WT-MT antigen-antibody pairs with explicit ΔΔG labels to guide the model to directly perceive the subtle binding intensity differences between similarity WT and MT structures, MCGLPPI is still able to give a reasonable predictive accuracy competitive to the specifically designed energy-based prediction tool FoldX. Furthermore, it also outperforms its atom- and residue-scale counterparts. In summary, the aforementioned experiments further demonstrate the advantages of our method and prove the flexibility and versatility of the CG-scale framework in handling various PPI property prediction tasks.

Supplementary Note 1 The version characteristics of MARTINI22 and MARTINI3

The MARTINI force field, particularly its MARTINI22 version[19], has been extensively employed for the coarse-grained (CG) representation of biomolecular systems[20,21]. However, as the usage of MARTINI22 has increased, several

shortcomings have been found, one of which is the tendency for some molecules to interact too strongly, primarily due to overly strong non-bonded interactions and the insufficient chemical space coverage brought by the relatively limited types and sizes of the CG beads[22,23]. To alleviate these limitations, Marrink et al. developed MARTINI3 force field[24], which features a rebalancing of all non-bonded interaction terms in the MARTINI model and introduces new bead types and labels, providing a richer and versatile representation of molecular interactions and dynamics. In our research, we explore the integration of the traditional MARTINI22 and the latest MARTINI3 CG-scale models with graph neural networks (GNNs) for predicting protein-protein interaction (PPI) overall properties.

Supplementary Note 2 Detailed curation process of the PDBbind-strict-dimer dataset

We retrieve 2852 protein–protein complexes with known binding affinity data in total from the PDBbind database (version 2020). Initially, we refine this dataset to include only the simplest types of complexes, which are composed of two protein components. We then select those samples that form a single PPI binding interface, as determined by their three-dimensional (3D) structural configurations. In cases where a two-component complex has multiple PPI binding interfaces, but these interfaces are structurally similar, we retain the sample and extract the structural information for the two proteins forming one representative binding interface. These filtering procedures result in a strict dataset of 1270 dimeric complexes (termed as the PDBbind-strict-dimer dataset).

Supplementary Note 3 The default hyper-parameters of involved approaches in comparison

We compare our CG-scale MCGLPPI framework with the counterpart GearNet-Edge which can function at the atom- or residue- scales[5,6]. We also incorporate an atom-scale advanced approach GVP-GNN[10], which is specifically designed to learn 3D macromolecular structures (esp., protein-protein complexes), into the complete comparison experiments. We give the basic default hyper-parameters of these involved approaches from original papers[5,6,10] as follows.

1. MCGLPPI (CG-scale, applicable to both MARTINI22- and MARTINI3-based versions): The detailed graph construction settings are provided in Methods of the manuscript. The default CG-scale graph convolutional layer number and hidden feature dimension are 6 and 256, respectively.
2. GearNet-Edge (atom-scale)[5,6]: The construction of protein graph structures for GearNet-Edge at the atom-scale is based on the radius edge: each atom node i will connect all other atom nodes within a 4.5Å-radius 3-dimensional (3D) sphere centered at i. The default graph convolutional layer number and hidden feature dimension are 6 and 128, respectively.
3. GearNet-Edge (residue-scale)[5,6]: The construction of protein graph structures for GearNet-Edge at the residue-scale is built based on three different types of distance-based or sequence-based edges.
    1) Radius edge: Analogous to those used in GearNet-Edge (atom-scale), the only difference is that the radius cutoff is set to 10 Å when wiring the neighboring

residue nodes.

2) KNN edge: Each residue node i connects other 10 nodes nearest to it, based on the Euclidean distance between their node position coordinates (and an extra 5 Å cutoff is imposed for limiting the minimum inter-residue distance).

3) Sequential edge: Two residue nodes will be wired if these two nodes have the relative positional difference N in the amino acid (AA) sequence of current protein. The pre-defined N includes 1, 2, and 3.

Besides, The default graph convolutional layer number and hidden feature dimension are 6 and 512, respectively.

4. GVP-GNN (atom-scale)[10]: Based on the same radius edge setting as GearNet-Edge at the atom-scale (4.5Å cutoff), the graph structure is constructed. GVP-GNN explicitly distinguishes scalars and vectors within the node and edge features. For the default hidden dimensions for node features, 100 (for scalars) and 16 (for vectors) are adopted. For the hidden dimensions of edge features, 32 (for scalars) and 1 (for vectors) are chosen. Besides, its default graph convolutional layer number is set to 5.

Supplementary Note 4 Investigation about the influence of hidden feature dimensions on overall performance of MCGLPPI

Because the default hidden feature dimensions of MCGLPPI and its atom- and residue-scale counterparts (i.e., GearNet-Edge) are 256, 128, and 512, respectively (the default graph convolutional layer number is the same: 6). In order to investigate the robustness of our MCGLPPI to the default dimensions of its atom- and residue-

scale counterparts, we conduct an extra experiment as follows.

Specifically, based on the experimental results on 915-subset of the PDBbind-strict-dimer dataset (see Table 1 of the manuscript), we can find the top predictive performance of atom- and residue-scale GearNet-Edge is both achieved at batch size equaling to 16. Therefore, we separately adjust the hidden feature dimension of MCGLPPI (MCGLPPI-M2 is used here) into 128 and 512 at the batch size 16 to conduct a fair comparison, and the results under the corresponding settings are recorded.

For the combination of hidden feature dimension 128 plus batch size 16, the results are: 0.587 (R_{P}), 2.088 (RMSE), 1.615 (MAE), 1625 (GPU (MB)), and 9818 (Total Time (s)). The corresponding results under the combination of hidden feature dimension 512 plus batch size 16 are: 0.579 (R_{P}), 2.079 (RMSE), 1.614 (MAE), 6658 (GPU (MB)), and 24,893 (Total Time (s)). We can observe that, after keeping the same hidden feature dimensions as the atom- and residue- counterparts, MCGLPPI still achieves the competitive predictive performance while preserving relatively lower computational cost (refer Table 1 of the manuscript, and the optimal results of MCGLPPI are achieved at a combination of hidden feature dimension 256 with batch size 64). This can validate the robustness of MCGLPPI to the hidden feature dimension, and further demonstrate the effectiveness of our CG-scale protein geometric representation learning framework.

Supplementary Note 5 More specific performance difference analysis across different MARTINI versions

When training from scratch, in the task of predicting PPI affinities on the PDBbind and ATLAS datasets, MCGLPPI-M3 outperforms MCGLPPI-M2. However, in the task of classifying complex interface structures on the MANY/DC dataset, the performance of MCGLPPI-M3 and MCGLPPI-M2 is comparable.

This variation in performance could be attributed to the fact that binding energy prediction is closely related to quantifiable physical parameters such as bead distance and interface charge[25]. MARTINI3 provides more appropriate bead segmentation and types than MARTINI22[24], leading to more precise descriptions of physical contacts and energy characterization. Consequently, the performance of MCGLPPI based on MARTINI3 tends to be superior in PPI affinity predictions. However, classifying PPI interfaces—involving biological or crystallographic classification—is more complex than free energy calculation, containing factors from the atomic level to the entire biological system level, which may explain the negligible performance differences between MCGLPPI-M2 and MCGLPPI-M3. Although MCGLPPI based on MARTINI3 outperforms its MARTINI2-based counterpart in terms of accuracy (in predicting binding affinities), the latter enjoys a slightly faster processing speed due to its reduced number of beads and bonds. This trade-off highlights the balance between computational efficiency and predictive accuracy within different versions of the MARTINI-based MCGLPPI models.

Furthermore, we also find that the use of DDIs-based pre-training not only improves the overall accuracy but also helps to minimize the performance differences between MCGLPPI-M2 and MCGLPPI-M3.

Supplementary Note 6 The potential extension of using corresponding force field parameters in other scales

In addition the CG-scale force field, all-atom force fields such as CHARMM[26] and AMBER[27] provide similar bonded and nonbonded parameters, which can be technically integrated with corresponding atom-scale GNNs to predict PPI properties. However, the number of edges in an atom-scale graph constructed from all-atom force field parameters exceeds those in a MARTINI-based CG-scale graph by more than three times. For instance, in the classical Barnase-Barstar protein complex, the AMBER99SB-ILDN force field[28] includes 1616 heavy atom bond parameters for graph edges, far exceeding the 495 and 521 bond parameters provided by the MARTINI22 and MARTINI3 force fields, respectively. Moreover, all-atom force fields offer a wider variety of atom types, angles, and dihedral parameters, indicating that GNN models based on all-atom fields are more complex and computationally demanding relative to their CG-scale counterparts. Furthermore, there are some simplified models where each protein residue is represented by a single bead, typically positioned at the $C\alpha$ location[29,30]. While these models are suited for simulating specific systems, they lose the granularity of the side chains and local structural nuances, facing difficulties in providing accurate and unbiased structural representations of more complex protein features when using one-bead-per-residue force field parameters. This limitation highlights the challenges involved in integrating residue-based force fields with corresponding GNNs. In total, the proposed MCGLPPI, which is based on the MARTINI force field, strikes a balance

between detailed structural representations of proteins and acceptable computational efficiency.

Supplementary Note 7 The definition of generation of a one-hot representation

"One-hot" representation refers to transforming the categorical scalar value from current feature (e.g., bead type of MARTINI22 or MARTINI3) into a unique binary representation, with the size equaling to the number of total categories appearing in current feature (e.g., 17 for bead type of MARTINI22 or 23 for bead type of MARTINI3). In this representation, the position corresponding to the current category is marked as 1, while all other positions are marked as 0.

Supplementary Note 8 Detailed description of allocation of sparse angular features to specific bead nodes

For each angular parameter generated by the MARTINI22 or MARTINI3 engines, its sine-cosine encoded feature will be allocated to specific bead node based on the following rules:

- The backbone angles (θ_{BBB}): For each θ_{BBB}, the corresponding encoded feature will be assigned to the second bead node of current BBB combination.
- The backbone-side chain angles (θ_{BBS}): For each θ_{BBS}, the corresponding encoded feature will be assigned to the third bead node of current BBS combination.
- The side chain angles (θ_{BSS}): For each θ_{BSS}, the corresponding encoded feature will be assigned to the third bead node of current BSS combination.

- The backbone dihedrals (Ψ_{BBBB}): For each Ψ_{BBBB}, the corresponding encoded feature will be assigned to the second bead node of current $BBBB$ combination.

Supplementary Note 9 The equations of the GearNet-Edge protein encoder

Concisely, GearNet-Edge introduces a line graph-augmented edge message passing strategy[31], which models the inter-edge relative positional relationship, to inject the additional structural information into the node representations for more effective protein geometric characteristics learning. The relevant equations are as follows:

$$\mathbf{h}_i^0 = \mathbf{f}_i \tag{1}$$

$$\mathbf{h}_i^l = \mathbf{h}_i^{l-1} + \mathbf{u}_i^l \tag{2}$$

$$\mathbf{u}_i^l = \sigma(BN\left(\sum_{r\in\mathcal{R}} W_r \sum_{j\in N_r(i)} \left(\mathbf{h}_j^{l-1} + MLP\left(\mathbf{m}_{(i,j,r)}^l\right)\right)\right)) \tag{3}$$

$$\mathbf{m}_{(j,i,r_1)}^0 = \mathbf{f}_{(j,i,r_1)} \tag{4}$$

$$\mathbf{m}_{(j,i,r_1)}^l = \sigma(BN(\sum_{r\in\mathcal{R}'} W_r' \sum_{(k,w,r_2)\in N_r'((j,i,r_1))} \mathbf{m}_{(k,w,r_2)}^{l-1})) \tag{5}$$

in which $\mathbf{f}_i$ and $\mathbf{f}_{(j,i,r)}$ are initial node and edge features, $\mathbf{h}_i^l$ and $\mathbf{u}_i^l$ are the hidden representation of node i at the l-th (graph convolutional) layer and aggregated neighboring information of node i at the l-th layer, respectively. For the calculation of $\mathbf{u}_i^l$ (equation (3)), σ, BN, W_r, $N_r(i)$, MLP, and $\mathbf{m}_{(i,j,r)}^l$ represent the Sigmoid activation function, Batch Normalization layer[32], edge-type-specific linear transformation matrix, 1-hop neighbors of node i with edge type r, multi-layer perception (MLP), and augmented edge feature based on the line graph at l-th layer, respectively. Furthermore, for the calculation of the augmented edge feature (equation

(5)), it follows the similar information aggregation mechanism as equation (3), which will aggregate the neighboring edges' information into the feature of the central edge based on a pre-defined line graph. For the description of the line graph construction among existing edges, please refer Zhang et al.[5] for detailed information.

Supplementary Note 10 The details about the CG-scale pre-training technique

For every protein domain-domain (DDI) complex curated from the 3DID dataset, we first transform it into a CG-scale complex graph following the same procedure as that for downstream complex transformations. These CG-scale DDI complex graphs will then be leveraged by a CG-scale diffusion denoising pre-training technique developed based on the atom-scale work[6], aiming to make the CG graph encoder aware the general DDI knowledge from these samples (these operations are applicable for both MARTINI22- and MARTINI3-based versions).

Specifically, by jointly modelling of coarse-grained protein complex conformation and underlying sequence information, the more comprehensive learning of CG DDI complex structures could be achieved. Based on this, the diffusion mechanism[33] is used to naturally add noise with varying magnitudes into the 3D coordinates and sequences of bead nodes within CG-scale DDI complex graphs to corrupt these graphs, and then to force the CG protein encoder to recover the original conformation and sequence for the complete joint modelling. The whole process is formulated as follows, the equations (6) and (7) represent the noising adding and denoising processes respectively.

$$q(D^{1:T}|D^0) = \prod_{t=1}^{T} q(D^t|D^{t-1}) \tag{6}$$

$$p_\theta(D^{0:T-1}|D^T) = \prod_{t=1}^{T} p_\theta(D^{t-1}|D^t) \tag{7}$$

in which the both processes can be decomposed into multiple time steps of noising adding or denoising following the rule of Markov chains (i.e., the total time steps are T). For each step t, $q(D^t|D^{t-1})$ and $p_\theta(D^{t-1}|D^t)$ define the current noising adding and denoising based on the parameterized CG graph encoder, respectively (in which D^t represents the status of the noised CG DDI complex (graph) at step t). Additionally, $q(D^t|D^{t-1})$ and $p_\theta(D^{t-1}|D^t)$ can be further decomposed into the CG conformation and bead sequence noising adding and denoising processes, to realize the aforementioned joint modelling (equations (8)-(9)).

$$q(D^t|D^{t-1}) = q(S^t|S^{t-1}) \cdot q(C^t|C^{t-1}) \tag{8}$$

$$p_\theta(D^{t-1}|D^t) = p_\theta(S^{t-1}|D^t) \cdot p_\theta(C^{t-1}|D^t) \tag{9}$$

in which C^t and S^t denote the status the of CG DDI complex conformation and bead sequence at step t, respectively.

For $q(S^t|S^{t-1})$ that is performed prior to the $q(C^t|C^{t-1})$ in each step of noising adding, all side chain bead nodes S (and their corresponding edges and features), which belong to the specified amino acids (AAs) to be masked, will be cropped out from the current CG DDI complex graph (to get a new cropped graph). This is for masking the sequence information that can indicate the AA types based on the pre-defined AA masking ratio value ρ^t. After that, the independent coordinate noise drawn based on the Gaussian distribution (controlled by the pre-defined distribution

variance value β^t) will be injected into the 3D coordinate of every bead node within current graph after the above cropping, to corrupt the overall CG-scale conformation for finishing current noising adding step.

With regards to the corresponding denoising process $p_\theta(D^{t-1}|D^t)$ for CG-scale conformation and sequence recovery, the cropped graph produced based on $q(D^t|D^{t-1})$ is sent to the CG graph encoder to generate the node-level geometric representations for current corrupted CG DDI complex. These representations will be further sent to the structural denoising network and sequential denoising network, for predicting 1) the CG-scale conformation change (brought by CG conformation noise adding) measured by the distance between pairwise bead nodes of edges within the cropped graph, and 2) the AA types which are masked in CG sequence noising adding, respectively. The mean square error (MSE, denoted as L^C) and the cross-entropy (CE, denoted as L^S) are chosen as the loss function to measure the respective differences between the recovered values and corresponding ground truths. The total loss to guide the pre-training model optimization can thus be formulated as follows, in which α is the loss weight to balance the both denoising process.

$$L^{pre-training} = \alpha L^S + (1-\alpha)L^C \tag{10}$$

For the aforementioned AA masking ratio ρ^t, distribution variance β^t, structural denoising network, and sequential denoising network, we follow the settings in ref. [6], we suggest referring the original paper for the detailed description. On top of this, the pre-training is performed based on the CG graph encoder on the curated CG-scale 3DID pre-training set under 200 epochs with $\alpha = 0.5$, followed by additional 50

epochs with $\alpha = 0.8$. In addition, another difference compared with the atom-scale work in ref. [6] is that, we do not incorporate the similar conformer generation mechanism, which aims to bring more conformation varieties of original proteins, into the CG-based diffusion training process, for which we find that it does not help to the further performance improvement for our CG-scale geometric learning framework.

Supplementary Note 11 Summary of implementation tools for MCGLPPI

Our basic program language is Python 3.9.18, on which Pytorch 1.12.1[34] and Torchdrug[35] 0.2.1 with a default random seed 0 are used to construct the overall framework of the MCGLPPI (including MCGLPPI-M2 and MCGLPPI-M3). For the CG structure and CG-scale force field parameter generation, the Python-based script martinize.py (https://cgmartini.nl/docs/downloads/tools/proteins-and-bilayers.html, version 2.4)[19] (for MARTINI22) as well as the Martinize2 and Vermouth programs[36] (for MARTINI3) are used for the transformation process. Besides, before the parameter generation, we adopt the pdbfixer tool (https://github.com/openmm/pdbfixer) to complete missing side-chains and convert non-natural amino acids to their natural counterparts.

Supplementary References

1 Baskaran K, Duarte J M, Biyani N, et al. A PDB-wide, evolution-based assessment of protein-protein interfaces[J]. BMC structural biology, 2014, 14: 1-11.
2 Duarte J M, Srebniak A, Schärer M A, et al. Protein interface classification by evolutionary analysis[J]. BMC bioinformatics, 2012, 13: 1-16.
3 Réau M, Renaud N, Xue L C, et al. DeepRank-GNN: a graph neural network framework to learn patterns in protein–protein interfaces[J]. Bioinformatics, 2023, 39(1): btac759.
4 Wang Z, Brand R, Adolf-Bryfogle J, et al. EGGNet, a generalizable geometric deep learning framework for protein complex pose scoring[J]. ACS omega, 2024, 9(7): 7471-7479.

5 Zhang Z, Xu M, Jamasb A R, et al. Protein Representation Learning by Geometric Structure Pretraining[C]//The Eleventh International Conference on Learning Representations.

6 Zhang Z, Xu M, Lozano A C, et al. Pre-training protein encoder via siamese sequence-structure diffusion trajectory prediction[J]. Advances in Neural Information Processing Systems, 2024, 36.

7 Zeng Y, Wei Z, Yuan Q, et al. Identifying B-cell epitopes using AlphaFold2 predicted structures and pretrained language model[J]. Bioinformatics, 2023, 39(4): btad187.

8 Kipf T N, Welling M. Semi-supervised classification with graph convolutional networks[J]. arXiv preprint arXiv:1609.02907, 2016.

9 Zhang, Y. & Skolnick, J. J. N. a. r. TM-align: a protein structure alignment algorithm based on the TM-score. 33, 2302-2309 (2005).

10 Jing B, Eismann S, Soni P N, et al. Equivariant graph neural networks for 3d macromolecular structure[J]. arXiv preprint arXiv:2106.03843, 2021.

11 Mirdita M, Schütze K, Moriwaki Y, et al. ColabFold: making protein folding accessible to all[J]. Nature methods, 2022, 19(6): 679-682.

12 Jumper J, Evans R, Pritzel A, et al. Highly accurate protein structure prediction with AlphaFold[J]. nature, 2021, 596(7873): 583-589.

13 Evans R, O’Neill M, Pritzel A, et al. Protein complex prediction with AlphaFold-Multimer[J]. biorxiv, 2021: 2021.10. 04.463034.

14 Sirin S, Apgar J R, Bennett E M, et al. AB-bind: antibody binding mutational database for computational affinity predictions[J]. Protein Science, 2016, 25(2): 393-409.

15 Yue Y, Li S, Wang L, et al. MpbPPI: a multi-task pre-training-based equivariant approach for the prediction of the effect of amino acid mutations on protein–protein interactions[J]. Briefings in Bioinformatics, 2023, 24(5): bbad310.

16 Schymkowitz J, Borg J, Stricher F, et al. The FoldX web server: an online force field[J]. Nucleic acids research, 2005, 33(suppl_2): W382-W388.

17 Liu X, Luo Y, Li P, et al. Deep geometric representations for modeling effects of mutations on protein-protein binding affinity[J]. PLoS computational biology, 2021, 17(8): e1009284.

18 Barlow K A, Ó Conchúir S, Thompson S, et al. Flex ddG: Rosetta ensemble-based estimation of changes in protein–protein binding affinity upon mutation[J]. The Journal of Physical Chemistry B, 2018, 122(21): 5389-5399.

19 De Jong D H, Singh G, Bennett W F D, et al. Improved parameters for the martini coarse-grained protein force field[J]. Journal of chemical theory and computation, 2013, 9(1): 687-697.

20 Marrink S J, Tieleman D P. Perspective on the Martini model[J]. Chemical Society Reviews, 2013, 42(16): 6801-6822.

21 Kmiecik S, Gront D, Kolinski M, et al. Coarse-grained protein models and their applications[J]. Chemical reviews, 2016, 116(14): 7898-7936.

22 Javanainen M, Martinez-Seara H, Vattulainen I. Excessive aggregation of membrane proteins in the Martini model[J]. PloS one, 2017, 12(11): e0187936.

23 Marrink S J, Monticelli L, Melo M N, et al. Two decades of Martini: Better beads, broader scope[J]. Wiley Interdisciplinary Reviews: Computational Molecular Science, 2023, 13(1): e1620.

24 Souza P C T, Alessandri R, Barnoud J, et al. Martini 3: a general purpose force field for coarse-grained molecular dynamics[J]. Nature methods, 2021, 18(4): 382-388.

25 Kortemme T, Baker D. A simple physical model for binding energy hot spots in protein–protein complexes[J]. Proceedings of the National Academy of Sciences, 2002, 99(22): 14116-14121.

26 Huang J, Rauscher S, Nawrocki G, et al. CHARMM36m: an improved force field for folded and intrinsically disordered proteins[J]. Nature methods, 2017, 14(1): 71-73.

27 Salomon-Ferrer R, Case D A, Walker R C. An overview of the Amber biomolecular simulation package[J]. Wiley Interdisciplinary Reviews: Computational Molecular Science, 2013, 3(2): 198-210.

28 Smith M D, Rao J S, Segelken E, et al. Force-field induced bias in the structure of Aβ21–30: A comparison of OPLS, AMBER, CHARMM, and GROMOS force fields[J]. Journal of chemical information and modeling, 2015, 55(12): 2587-2595.

29 Bahar I, Jernigan R L. Inter-residue potentials in globular proteins and the dominance of highly specific hydrophilic interactions at close separation[J]. Journal of molecular biology, 1997, 266(1): 195-214.

30 Clementi C, Nymeyer H, Onuchic J N. Topological and energetic factors: what determines the structural details of the transition state ensemble and "en-route" intermediates for protein folding? An investigation for small globular proteins[J]. Journal of molecular biology, 2000, 298(5): 937-953.

31 Harary F, Norman R Z. Some properties of line digraphs[J]. Rendiconti del circolo matematico di palermo, 1960, 9: 161-168.

32 Santurkar S, Tsipras D, Ilyas A, et al. How does batch normalization help optimization?[J]. Advances in neural information processing systems, 2018, 31.

33 Ho J, Jain A, Abbeel P. Denoising diffusion probabilistic models[J]. Advances in neural information processing systems, 2020, 33: 6840-6851.

34 Paszke A, Gross S, Massa F, et al. Pytorch: An imperative style, high-performance deep learning library[J]. Advances in neural information processing systems, 2019, 32.

35 Zhu Z, Shi C, Zhang Z, et al. Torchdrug: A powerful and flexible machine learning platform for drug discovery[J]. arXiv preprint arXiv:2202.08320, 2022.

36 Kroon P C, Grunewald F, Barnoud J, et al. Martinize2 and vermouth: unified framework for topology generation. Elife 12[J]. RP90627, 2023.
