# Supplementary material for: Integration of molecular coarse-grained model into geometric representation learning framework for protein-protein complex property prediction
Source: Nat Commun. 2024 Nov 7;15:9629. doi: 10.1038/s41467-024-53583-w (PMC11544137; doi:10.1038/s41467-024-53583-w)
Supplement: Supplementary file 2 — Reporting Summary [file 41467_2024_53583_MOESM2_ESM.pdf]

nature portfolio

Corresponding author(s): Shan He

Last updated by author(s): Sep 16, 2024

Reporting Summary

Nature Portfolio wishes to improve the reproducibility of the work that we publish. This form provides structure for consistency and transparency in reporting. For further information on Nature Portfolio policies, see our Editorial Policies and the Editorial Policy Checklist.

Statistics

For all statistical analyses, confirm that the following items are present in the figure legend, table legend, main text, or Methods section.

n/a	Confirmed	
☐	☒	The exact sample size (*n*) for each experimental group/condition, given as a discrete number and unit of measurement
☐	☒	A statement on whether measurements were taken from distinct samples or whether the same sample was measured repeatedly
☒	☐	The statistical test(s) used AND whether they are one- or two-sided<br>*Only common tests should be described solely by name; describe more complex techniques in the Methods section.*
☒	☐	A description of all covariates tested
☒	☐	A description of any assumptions or corrections, such as tests of normality and adjustment for multiple comparisons
☒	☐	A full description of the statistical parameters including central tendency (e.g. means) or other basic estimates (e.g. regression coefficient) AND variation (e.g. standard deviation) or associated estimates of uncertainty (e.g. confidence intervals)
☒	☐	For null hypothesis testing, the test statistic (e.g. *F*, *t*, *r*) with confidence intervals, effect sizes, degrees of freedom and *P* value noted<br>*Give P values as exact values whenever suitable.*
☒	☐	For Bayesian analysis, information on the choice of priors and Markov chain Monte Carlo settings
☒	☐	For hierarchical and complex designs, identification of the appropriate level for tests and full reporting of outcomes
☐	☒	Estimates of effect sizes (e.g. Cohen's *d*, Pearson's *r*), indicating how they were calculated

Our web collection on statistics for biologists contains articles on many of the points above.

Software and code

Policy information about availability of computer code

Data collection	Code to run MCGLPPI (Version 1.0) is available at https://github.com/arantir123/MCGLPPI.
Data analysis	Code to reproduce the analysis (Version 1.0) is available at https://github.com/arantir123/MCGLPPI.

For manuscripts utilizing custom algorithms or software that are central to the research but not yet described in published literature, software must be made available to editors and reviewers. We strongly encourage code deposition in a community repository (e.g. GitHub). See the Nature Portfolio guidelines for submitting code & software for further information.

Data

Policy information about availability of data

All manuscripts must include a data availability statement. This statement should provide the following information, where applicable:

- Accession codes, unique identifiers, or web links for publicly available datasets
- A description of any restrictions on data availability
- For clinical datasets or third party data, please ensure that the statement adheres to our policy

All datasets analyzed are freely available through the original sources:
· 3DID: https://3did.irbbarcelona.org/
· PDBbind: http://www.pdbbind.org.cn/
· ATLAS: https://pubmed.ncbi.nlm.nih.gov/28160322/

· MANY and DC: https://www.eppic-web.org/ewui/#downloads
· AB-bind: https://www.ncbi.nlm.nih.gov/pmc/articles/PMC4815335/
Easing access, we re-packaged all data at https://github.com/arantir123/MCGLPPI. When using those data, please quote and consult the authors of the original datasets. Source data are provided within this paper.

Research involving human participants, their data, or biological material

Policy information about studies with human participants or human data. See also policy information about sex, gender (identity/presentation), and sexual orientation and race, ethnicity and racism.

Reporting on sex and gender	The present study did not involve human participants.
Reporting on race, ethnicity, or other socially relevant groupings	The present study did not involve human participants.
Population characteristics	The present study did not involve human participants.
Recruitment	The present study did not involve human participants.
Ethics oversight	The present study did not involve human participants.

Note that full information on the approval of the study protocol must also be provided in the manuscript.

Field-specific reporting

Please select the one below that is the best fit for your research. If you are not sure, read the appropriate sections before making your selection.

☒ Life sciences ☐ Behavioural & social sciences ☐ Ecological, evolutionary & environmental sciences

For a reference copy of the document with all sections, see nature.com/documents/nr-reporting-summary-flat.pdf

Life sciences study design

All studies must disclose on these points even when the disclosure is negative.

Sample size	In this study, we organized and refined data from five distinct datasets. Firstly, the pre-training dataset comprises a total of 41,663 domain-domain interaction (DDI) structure samples from latest 3DID database. Secondly, from the PDBbind dataset, we extracted 1,270 protein-protein complexes that display strict dimer structures, each annotated with binding affinity labels (ΔG). Thirdly, we processed the ATLAS dataset, which includes TCR-pMHC structures pertinent to cell-mediated immunity, along with their associated binding affinities. After discarding invalid entries, correcting errors, and standardizing labels, we were left with 531 unique structures, all with ΔG annotations. Fourth, we utilized the MANY and DC datasets, which contain 5,739 and 161 dimer samples, respectively. These samples fall into two broad categories based on the nature of their interfaces: biological or crystal. Lastly, the AB-bind dataset was used, in which 1,101 wild-type (WT) mutant (MT) complex pairs with binding affinity change caused by amino acid mutations labels (ΔΔG) are included.
Data exclusions	For the 3DID dataset used for model pre-training, we removed any DDI samples from the 3DID dataset that are identical to those present in our downstream benchmark datasets. In the PDBbind dataset, we refined this dataset to include only the simplest types of complexes, which are composed of two protein components. We then selected those samples that form a single PPI binding interface, as determined by their three-dimensional (3D) structural configurations. In cases where a two-component complex has multiple PPI binding interfaces, but these interfaces are structurally similar, we retained the sample and extracted the structural information for the two proteins forming one representative binding interface. These filtering procedures resulted in a strict dataset of 1270 dimeric complexes (termed as the PDBbind-strict-dimer dataset).
Replication	We performed tenfold cross-validation and reported the average results of these independent runs.
Randomization	In this study, we used two allocation methods:<br>1. Random Allocation: Each dataset was randomly shuffled to make different sample points enter each fold.<br>2. Homology Reduction Allocation: Samples were allocated based on structural similarity to minimize structural similarity between the training and test sets.
Blinding	No blinding was included in this study because the data is publicly available and the analysis was automated, eliminating the possibility of human intervention introducing bias.

Reporting for specific materials, systems and methods

We require information from authors about some types of materials, experimental systems and methods used in many studies. Here, indicate whether each material, system or method listed is relevant to your study. If you are not sure if a list item applies to your research, read the appropriate section before selecting a response.

Materials & experimental systems

n/a	Involved in the study
☒	☐ Antibodies
☒	☐ Eukaryotic cell lines
☒	☐ Palaeontology and archaeology
☒	☐ Animals and other organisms
☒	☐ Clinical data
☒	☐ Dual use research of concern
☒	☐ Plants

Methods

n/a	Involved in the study
☒	☐ ChIP-seq
☒	☐ Flow cytometry
☒	☐ MRI-based neuroimaging

Plants

Seed stocks	N/A
Novel plant genotypes	N/A
Authentication	N/A
